# High-Performance Thin-Film Transistors with ZnO:H/ZnO Double Active Layers Fabricated at Room Temperature

**DOI:** 10.3390/nano13081422

**Published:** 2023-04-20

**Authors:** Daoqin Wang, Zongjin Jiang, Linhan Li, Deliang Zhu, Chunfeng Wang, Shun Han, Ming Fang, Xinke Liu, Wenjun Liu, Peijiang Cao, Youming Lu

**Affiliations:** Shenzhen Key Laboratory of Special Functional Materials, Guangdong Research Centre for Interfacial Engineering of Functional Materials, College of Materials Science and Engineering, Shenzhen University, Shenzhen 518000, China

**Keywords:** thin-film transistors, ZnO:H/ZnO double active layers, RF magnetron sputtering, room temperature

## Abstract

H doping can enhance the performance of ZnO thin-film transistors (TFTs) to a certain extent, and the design of double active layers is an effective way to further improve a device’s performance. However, there are few studies on the combination of these two strategies. We fabricated TFTs with ZnO:H (4 nm)/ZnO (20 nm) double active layers by magnetron sputtering at room temperature, and studied the effect of the hydrogen flow ratio on the devices’ performance. ZnO:H/ZnO-TFT has the best overall performance when H_2_/(Ar + H_2_) = 0.13% with a mobility of 12.10 cm^2^/Vs, an on/off current ratio of 2.32 × 10^7^, a subthreshold swing of 0.67 V/Dec, and a threshold voltage of 1.68 V, which is significantly better than the performance of single active layer ZnO:H-TFTs. This exhibits that the transport mechanism of carriers in double active layer devices is more complicated. On one hand, increasing the hydrogen flow ratio can more effectively suppress the oxygen-related defect states, thus reducing the carrier scattering and increasing the carrier concentration. On the other hand, the energy band analysis shows that electrons accumulate at the interface of the ZnO layer close to the ZnO:H layer, providing an additional path for carrier transport. Our research exhibits that the combination of a simple hydrogen doping process and double active layer construction can achieve the fabrication of high-performance ZnO-based TFTs, and that the whole room temperature process also provides important reference value for the subsequent development of flexible devices.

## 1. Introduction

At present, the traditional silicon-based active layer thin film transistor (TFT) can no longer meet the requirements of high-quality displays. Metal oxide TFTs (MOTFTs) have the advantages of high carrier mobility, stable and uniform performance, and high transmittance, etc., so MOTFTs show great application prospects in CMOS sensors, flat panel displays, and other fields [1,2,3,4]. Among the metal oxide active layer materials, ZnO has the characteristics of being cheap, abundant, having a simple process, and being non-toxic, and can be prepared at low temperatures and applied to flexible substrates to manufacture flexible electronic devices. It conforms to the trend of flexible displays and wearable devices, therefore attracting the attention of many scholars [5,6,7,8,9,10]. However, ZnO thin films generally exhibit a polycrystalline state with a large number of intrinsic defects, and the carriers are scattered and trapped by high-density intrinsic defect states and grain boundaries during their transportation, resulting in poor performance of ZnO-TFTs.

Doping is an effective way to improve the device performance of ZnO-TFTs [11,12,13], among which hydrogen doping possesses the advantages of simple process, environmental friendliness, and low cost, etc. [14,15,16,17]. A small amount of H doping can increase the on-current (*I*_on_) of ZnO-TFTs, as well as improve device mobility and bias stability to a certain extent by reducing the interface trap density and increasing the carrier concentration. However, it is easy for hydrogen doping to exceed the appropriate ratio; more hydrogen doping will lead to a too high carrier concentration, significantly increasing the off-current (*I*_off_) and changing the device to be in depletion mode, and even making the device show metallic conductivity. Thus, the hydrogen doping method has certain limitations. It is also an effective method to enhance a device’s performance by using the double active layer structure to fabricate TFTs, but the mechanism of improving TFT performance with double active layers is still controversial [18,19,20,21,22].

We considered combining hydrogen doping with the design of a double active layer structure to prepare ZnO:H/ZnO double active layer TFTs by RF magnetron sputtering to further improve the devices’ performance. The related research was only reported by Abliz et al. [21]; however, their hydrogen doping was realized by surface plasma treatment after film deposition, which is a secondary process. In addition, their work involved substrate heating during sputtering and annealing after film deposition. The above complex process steps and relatively high process temperature are not conducive to the industrial application of devices, especially in the field of flexible electronics. Thus, we firstly chose a simpler atmosphere doping method, directly introducing H_2_ into the sputtering gas to achieve H doping. Secondly, we tried to fabricate ZnO:H/ZnO-TFTs without heat treatment in the whole process. Moreover, the physical mechanism of the double active layer structure to improve the devices’ performance was analyzed. The high-performance ZnO:H/ZnO-TFTs achieved by a simple hydrogen doping process at room temperature have huge potential for large-scale and low-cost flexible electronics. 

## 2. Experimental Section

**Figure 1:**
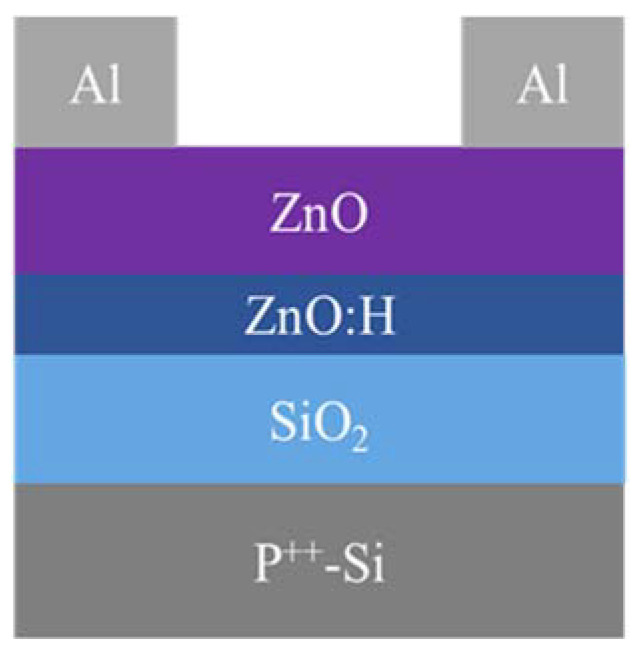
The device structure of bottom-gate top-contact ZnO:H/ZnO-TFT.

Figure 1 exhibits the device structure of bottom-gate top-contact ZnO:H/ZnO-TFT; a 20 nm ZnO back channel layer and 4 nm ZnO:H front channel layer were, respectively, deposited by RF magnetron sputtering on SiO_2_ (100 nm)/P^++^-Si substrate, where SiO_2_ was used as the insulator layer and P^++^-Si as the gate electrode. The substrate was ultrasonically cleaned with acetone, ethyl alcohol, and deionized water for 15 min, respectively, then dried with N_2_ before deposition. The double active layers were prepared at room temperature (without intentionally heating the substrates) with 40 W power and 0.5 Pa working pressure, where 75 sccm pure Ar gas flow was used for ZnO deposition, while 75 sccm total gas flow within the flow ratio of H_2_/(Ar + H_2_) = 0% to 0.33%, i.e., the hydrogen flow was 0–0.25 sccm with intervals of 0.05 sccm, was used for ZnO:H deposition. The deposition rate of ZnO was around 5 nm/min. Then, the devices were fabricated by using a shadow mask with a channel length of 100 μm and width of 1500 μm, and 100 nm Al with a deposition rate of 18 nm/min was prepared as source/drain electrodes by thermal evaporation.

The secondary ion mass spectrometry (SIMS) depth profiles of ZnO:H thin films were measured by a secondary ion mass spectrometer (ION TOF-SIMS 5). The structure of ZnO:H/ZnO films was studied by grazing incidence X-ray diffraction (GIXRD, Rigaku SmartLab). The oxygen chemical states in ZnO:H films were analyzed by X-ray photoelectron spectroscopy (XPS, Thermo Microlab 350). The band structures of ZnO and ZnO:H films were analyzed by a UV-Vis spectrophotometer (Shimadzu UV-2450PC) and an ultraviolet photoelectron spectrometer (UPS, Thermo ESCALAB 250Xi). The electrical performance of ZnO:H/ZnO-TFTs was examined with a semiconductor parameter analyzer (Keithley 2614B) at room temperature in a dark environment. 

## 3. Results and Discussion

**Figure 2:**
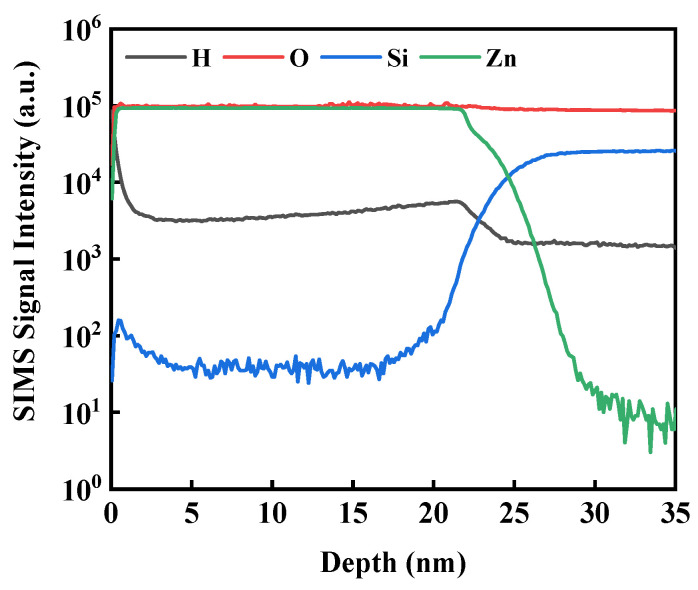
SIMS depth profiles of 20 nm ZnO:H thin film deposited at H_2_/(Ar + H_2_) = 0.13%.

Figure 2 exhibits the SIMS depth profiles of 20 nm ZnO:H thin film deposited on P^++^-Si/SiO_2_ substrate at H_2_/(Ar + H_2_) = 0.13%. Hydrogen was found to exist in the ZnO thin film, and all elements were uniformly distributed with depth except the abrupt changes on the surface and at 20 nm. It exhibited the feasibility of H doping in ZnO films by directly introducing H_2_ into the sputtering gas.

**Figure 3:**
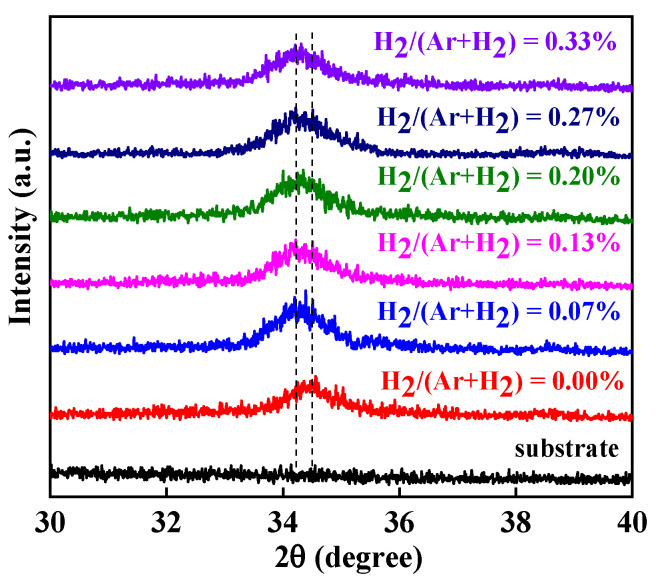
GIXRD curves of ZnO:H/ZnO films prepared with different H_2_/(Ar + H_2_).

Since the active layers were thin, GIXRD was used to analyze the structure of the ZnO:H/ZnO films, where the front channel layers were prepared with different H_2_/(Ar + H_2_) from 0% to 0.33%. As shown in Figure 3, there were only ZnO (002) diffraction peaks for all samples, indicating that all the ZnO:H/ZnO films were hexagonal wurtzite structures with c-axis preferential orientation. Combined with SIMS experimental results, H doping in ZnO lattice was realized. The weak intensity and wide full-width at half-maximum (FWHM) of the diffraction peaks exhibit that the crystalline quality of all films was poor and the grain size was small, which is possibly because the substrates were not intentionally heated. Additionally, the FWHM of the film without hydrogen doping was narrower than those of all the H-doped films, showing that the addition of H_2_ in the sputtering gas reduced the grain size and increased the grain boundaries, which may be due to the influence of H doping on the nucleation process in film crystallization [14]. We can see from the dotted line mark in Figure 3 that the (002) diffraction peak slightly shifted to a lower angle with increasing H_2_/(Ar + H_2_). It is known from the Bragg equation that this was caused by the increase in interplanar spacing. Since part of the doped H existed in the form of hydrogen interstitials (H_i_), H_i_ were located at the center of Zn-O bonds, resulting in the increase in interplanar spacing of c-axis direction [23]. The lattice distortion induced by the H incorporation may also be the reason for the larger FWHM of the H-doped ZnO:H/ZnO films [14]. In comparison, the diffraction peak of a single ZnO:H active layer shifted more obviously with the hydrogen flow ratio [17]; this may be due to the fact that the thickness of a single ZnO:H active layer was several tens of nanometers, much thicker than the 4 nm ZnO:H film in double active layers in this research. It must be mentioned that the increase in grain boundaries and hydrogen-related defects increase the scattering of carriers in the front channel layer, which adversely affects the devices’ performance.

**Figure 4:**
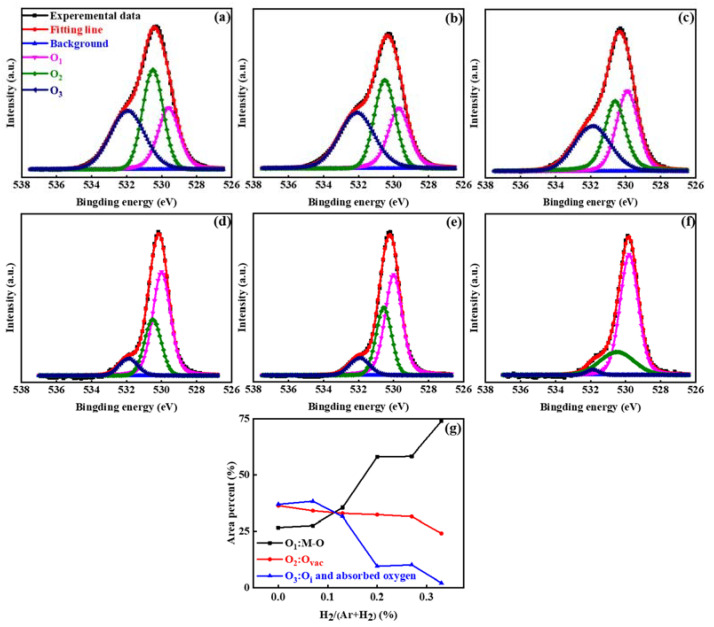
XPS spectra of O 1s with different H_2_/(Ar + H_2_): (**a**) 0%, (**b**) 0.07%, (**c**) 0.13%, (**d**) 0.20%, (**e**) 0.27%, (**f**) 0.33%; (**g**) the proportion variation of three oxygen components with H_2_ flow ratio.

To analyze the effect of H doping on oxygen-related defects in ZnO films, the O 1s energy spectra of ZnO:H films with different H_2_/(Ar + H_2_) were obtained by XPS measurement and are shown in Figure 4. The O 1s energy spectra were calibrated by using 285 eV C 1s binding energy as the standard in order to compare the measured O 1s binding energy with the data from the NIST database and other references. As exhibited in Figure 4a–f, the fitting results consist of three components of oxygen in varying chemical states [24,25,26,27]. The fitting peak at 529.8 ± 0.2 eV is related to the O^2−^ in metal oxide, representing the lattice oxygen existing in the Zn-O bonds, marked as O_1_. The fitting peak at 530.7 ± 0.2 eV is associated with O^2−^ ions in the oxygen-deficient regions within the matrix of ZnO, and the peak intensity reflects the change of oxygen vacancy (V_O_) content, marked as O_2_. The fitting peak at 531.9 ± 0.2 eV is attributed to the loosely bound oxygen in ZnO, including oxygen interstitials (O_i_) and adsorbed oxygen [28], marked as O_3_. From the integrated intensities of three fitting peaks, the proportion of three oxygen components can be obtained according to Equation (1).

(1) $$O_{x}=\frac{O_{x}}{O_{1}+O_{2}+O_{3}}\times 100\%$$

From Figure 4g, it is found that the proportion of lattice oxygen was much lower and the proportion of oxygen-related defects was much higher in the undoped ZnO film. With the increase in H_2_/(Ar + H_2_), the former significantly increased and the latter obviously decreased. The decrease in V_O_ is attributed to the formation of H-V_O_ complexes (H_O_), and the decrease in O_i_ and adsorbed oxygen is due to the passivation effect of hydrogen on the films’ surface and interface [29,30,31,32,33]. The change in oxygen-related defect states with H_2_ flow ratio will certainly affect the devices’ performance, which will be discussed later. 

**Figure 5:**
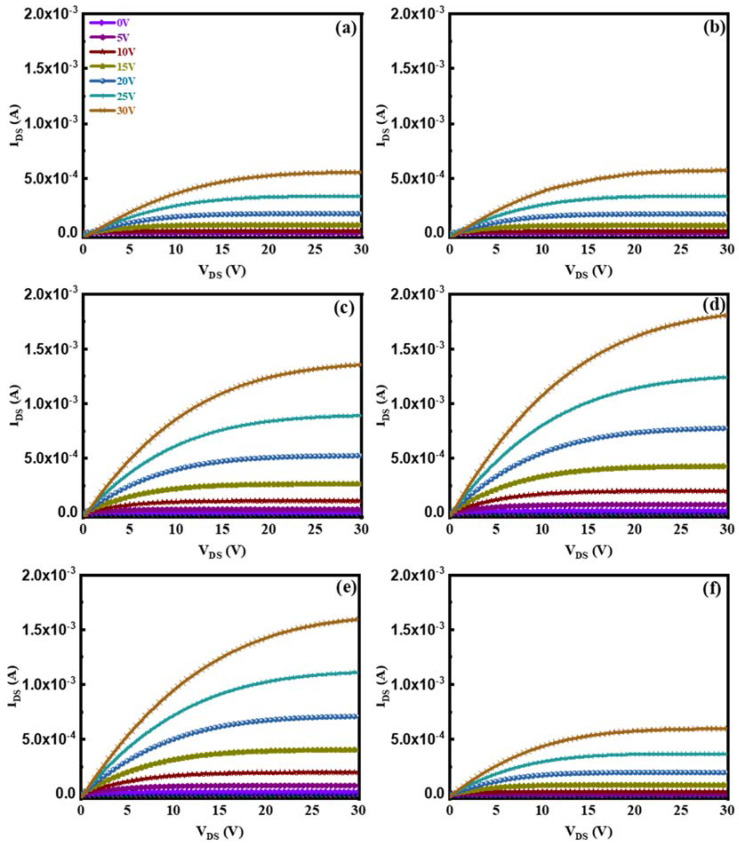
The output curves of ZnO:H/ZnO-TFTs fabricated with different H_2_/(Ar + H_2_): (**a**) 0%, (**b**) 0.07%, (**c**) 0.13%, (**d**) 0.20%, (**e**) 0.27%, (**f**) 0.33%.

The output curves of our devices fabricated with different H_2_ flow ratios are shown in Figure 5. All the devices exhibit n-type TFT output characteristics without any current crowding, reflecting good ohmic contact between the active layers and the source/drain electrodes. The output curves of all ZnO:H/ZnO-TFTs show obvious pinch-off and source-drain current saturation, indicating that the devices have good switching characteristics and reach the saturation mode under test conditions. Compared with the undoped device, all H-doped devices exhibit higher *I*_on_. With increasing H_2_/(Ar + H_2_), *I*_on_ first increases and then decreases. When *V*_DS_ = 30 V and *V*_GS_ = 30 V, *I*_on_ is 5.55 × 10^−4^ A for the undoped device; it reaches a maximum value of 1.81 × 10^−3^ A for the device with H_2_/(Ar + H_2_) = 0.20%, more than three times higher than that of the former. Additionally, it decreases to 5.97 × 10^−4^ A for the device with H_2_/(Ar + H_2_) = 0.33%, similar to that of the undoped device.

**Figure 6:**
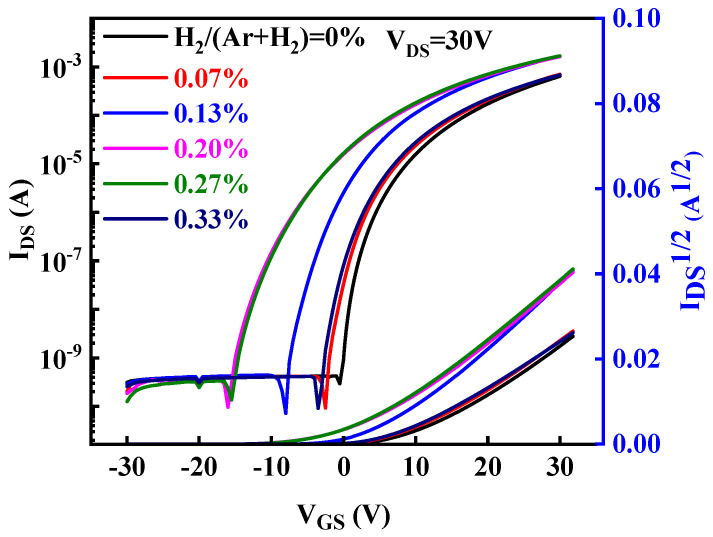
The transfer curves and the corresponding *I*_DS_^1/2^-*V*_GS_ curves of ZnO:H/ZnO-TFTs fabricated with different H_2_/(Ar + H_2_).

Figure 6 shows the transfer curves and the corresponding *I*_DS_^1/2^-*V*_GS_ curves of our devices fabricated with different H_2_ flow ratios. The characteristic parameters of the devices extracted from Figure 6 are listed in Table 1, including saturation mobility (*μ*_sat_), on/off ratio (*I*_on_/*I*_off_), threshold voltage (*V*_th_), and sub-threshold swing (*SS*). As shown in Table 1, all devices exhibit good comparable *SS* values. With the increase in H_2_/(Ar + H_2_), *μ*_sat_ and *I*_on_/*I*_off_ increase first and then decrease, while *V*_th_ shows the opposite change. That is, the devices’ performance first becomes better and then worse. When H_2_/(Ar + H_2_) = 0.13%, ZnO:H/ZnO-TFT with the best performance parameters was obtained: *μ*_sat_ = 12.10 cm^2^/Vs, *I*_on_/*I*_off_ = 2.32 × 10^7^, *SS* = 0.67 V/Dec, *V*_th_ = 1.68 V. With the further increase in H_2_/(Ar + H_2_), the devices’ performance became worse. 

According to the above characterization, on the one hand, the oxygen-related defects in the front channel layer (ZnO:H film) decrease with the increase in H_2_/(Ar + H_2_), i.e., the trap sites and electron scattering at the dielectric/active layer interface decrease, which facilitates the transport of electrons in the channel. Moreover, the decrease in adsorbed oxygen and O_i_ as acceptors and the increase in H_O_ and H_i_ as donors lead to an increase in carrier concentration in the channel [29,30,31,32,33]. The above factors improve the performance of ZnO:H/ZnO-TFT. On the other hand, hydrogen doping induces the increase in grain boundaries and hydrogen-related defects in ZnO:H film, and the scattering of electrons is enhanced in the channel. These factors deteriorate the performance of ZnO:H/ZnO-TFT. When H_2_/(Ar + H_2_) is relatively small, the former factors dominate, and the devices’ performance becomes better with the increase in H_2_ flow ratio. When the H_2_ flow rate is relatively large, the latter factors dominate, and the device performance becomes worse with increasing H_2_ flow ratio.

**Figure 7:**
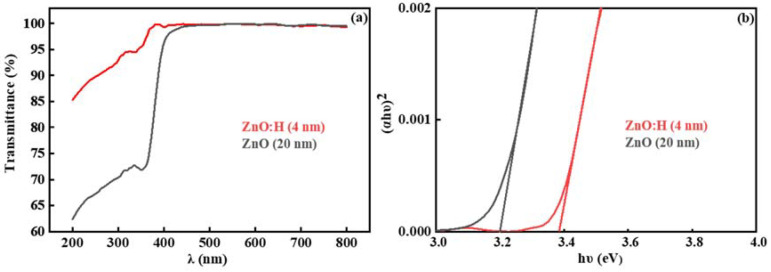
(**a**) UV-Vis transmission spectra and (**b**) the function relationship curves of (*αhν*)^2^-*hν* of ZnO:H prepared at H_2_/(Ar + H_2_) = 0.13% and ZnO channel layers.

**Figure 8:**
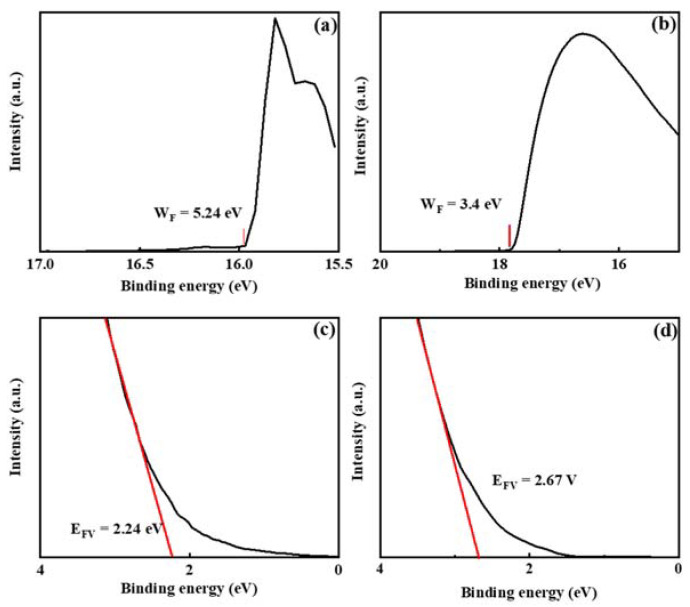
UPS results near the cut-off edge of high binding energy for (**a**) ZnO and (**b**) ZnO:H prepared at H_2_/(Ar + H_2_) = 0.13%; UPS results near the cut-off edge of low binding energy for (**c**) ZnO and (**d**) ZnO:H prepared at H_2_/(Ar + H_2_) = 0.13%.

**Figure 9:**
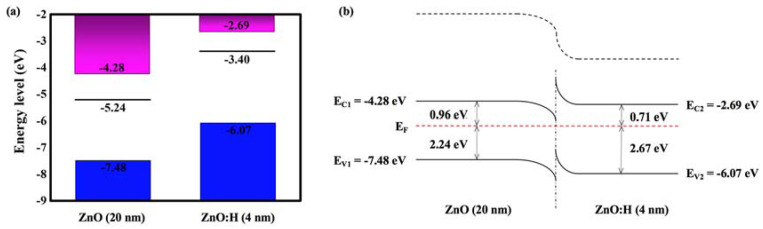
The band structures of ZnO and ZnO:H prepared at H_2_/(Ar + H_2_) = 0.13%: (**a**) before contact and (**b**) after contact.

As exhibited in Table 1, compared with the ZnO:H single active layer device with optimal performance fabricated at H_2_/(Ar + H_2_) = 0.10% [17], the ZnO:H/ZnO double active layer devices in this paper have significantly improved performance. In the previous study, the single-layer ZnO:H films were much thicker (50 nm), and the number of carriers in the active layers increased significantly due to H doping. When H_2_/(Ar + H_2_) = 0.13%, *I*_off_ increased dramatically, *I*_on_/*I*_off_ decreased dramatically, and the devices’ switching performance deteriorated. However, in ZnO:H/ZnO double active layer devices, the number of carriers is relatively limited with the increase in H_2_/(Ar + H_2_) due to the much thinner ZnO:H front channel layers. Therefore, we can increase the experimental range of the H_2_ flow ratio to reduce the carrier scattering by suppressing the oxygen-related defects more effectively. Moreover, due to the adjustment of source-drain resistance by ZnO back channel layers, better performance is obtained in ZnO:H/ZnO-TFTs compared with ZnO:H-TFTs. In addition, the better performance in ZnO:H/ZnO-TFTs may also be related to the interface between ZnO:H and ZnO. It is suggested that due to their different band structures, there exists a potential barrier at the ZnO:H/ZnO interface, which provides an extra carrier transport path for ZnO:H/ZnO-TFTs. In order to explore the carrier transport mechanism in the double active layer devices, the UV-Vis transmission spectra of 4 nm ZnO:H film prepared with H_2_/(Ar + H_2_) = 0.13% and 20 nm ZnO film deposited on the sapphire substrates were measured, and the corresponding results are exhibited in Figure 7a. The films’ optical bandgaps (*E*_g_) can be further obtained by the function relationship curves of (*αhν*)^2^-*hν*, as exhibited in Figure 7b. The obtained *E*_g_ was 3.20 and 3.38 eV for ZnO and ZnO:H films, respectively. Figure 8 shows the UPS results of ZnO and ZnO:H films. The difference between the excitation source energy (He I: 21.2 eV) and the cut-off value of high binding energy is the work function (*W*_F_) of the material [18]. For ZnO and ZnO:H films, *W*_F_ = 5.24 and 3.40 eV, respectively. The slope of the cut-off edge of low binding energy represents the energy difference (*E*_FV_) between the Fermi level (*E*_F_) and the valence band top (*E*_V_) [18]. *E*_FV_ = 2.24 and 2.67 eV for ZnO and ZnO:H films, respectively. Based on the above results, the band structures of ZnO (20 nm) and ZnO:H (4 nm) thin films before contact can be obtained, as shown in Figure 9a. Due to the high lattice matching between ZnO and ZnO:H films, we ignore the influence of surface states on the contact barrier. After contact, due to their different Fermi levels, the electrons in ZnO:H flow to ZnO, and the generated built-in electric field causes the band bending at the interface. The band structure when the equilibrium state is reached is exhibited in Figure 9b. According to Figure 9b, the electrons flowing from ZnO:H to ZnO accumulate at the interface of the ZnO layer close to the ZnO:H layer, and the existence of energy valley limits the dimension of electron movement, providing an additional path for carrier transport in ZnO:H/ZnO-TFTs. Due to the existence of double transport interfaces between dielectric and active layers and between ZnO:H and ZnO layers, the device performance of ZnO:H/ZnO-TFTs has been improved significantly compared with that of ZnO:H-TFTs.

## 4. Conclusions

Our research shows that the combination of a simple hydrogen doping process and double active layer design can significantly improve the performance of ZnO-based TFTs. H doping in the front channel layer can effectively reduce the scattering of carriers while increasing the carrier concentration. More importantly, the interface between ZnO and ZnO:H provides an additional path for carrier transport. The optimized device exhibits *μ*_sat_ of 12.10 cm^2^/Vs, *I*_on_/*I*_off_ of 2.32 × 10^7^, *SS* of 0.67 V/Dec, and *V*_th_ of 1.68 V. In this study, there is no heat treatment operation in the whole process of device fabrication, which is helpful for the devices’ subsequent application in the field of flexible electronics.

## Figures and Tables

**Table 1:** The device characteristics of ZnO:H/ZnO-TFTs fabricated with different H_2_/(Ar + H_2_) and ZnO:H-TFT fabricated at H_2_/(Ar + H_2_) = 0.10%.

H_2_/(Ar + H_2_) (%)	*μ*_sat_ (cm^2^/Vs)	*V*_th_ (V)	*I*_on_/*I*_off_	*SS* (V/Dec)
0	6.52	5.68	2.19 × 10^6^	0.78
0.07	6.53	5.41	7.65 × 10^6^	0.68
0.13	12.10	1.68	2.32 × 10^7^	0.67
0.20	9.88	0.26	1.76 × 10^7^	0.99
0.27	9.04	1.24	1.47 × 10^7^	1.07
0.33	5.47	5.35	7.52 × 10^6^	0.84
ZnO:H-TFT [17]	5.17	6.03	4.98 × 10^6^	0.76

## Data Availability

Data is available from the corresponding authors upon request.
